# Online transcranial Doppler ultrasonographic control of an onscreen keyboard

**DOI:** 10.3389/fnhum.2014.00199

**Published:** 2014-04-22

**Authors:** Jie Lu, Khondaker A. Mamun, Tom Chau

**Affiliations:** ^1^Bloorview Research Institute, Holland Bloorview Kids Rehabilitation HospitalToronto, ON, Canada; ^2^Institute of Biomaterials and Biomedical Engineering, University of TorontoToronto, ON, Canada

**Keywords:** TCD, BCI, middle cerebral artery, hemodynamic response, lateralization, communication

## Abstract

Brain-computer interface (BCI) systems exploit brain activity for generating a control command and may be used by individuals with severe motor disabilities as an alternative means of communication. An emerging brain monitoring modality for BCI development is transcranial Doppler ultrasonography (TCD), which facilitates the tracking of cerebral blood flow velocities associated with mental tasks. However, TCD-BCI studies to date have exclusively been offline. The feasibility of a TCD-based BCI system hinges on its online performance. In this paper, an online TCD-BCI system was implemented, bilaterally tracking blood flow velocities in the middle cerebral arteries for system-paced control of a scanning keyboard. Target letters or words were selected by repetitively rehearsing the spelling while imagining the writing of the intended word, a left-lateralized task. Undesired letters or words were bypassed by performing visual tracking, a non-lateralized task. The keyboard scanning period was 15 s. With 10 able-bodied right-handed young adults, the two mental tasks were differentiated online using a Naïve Bayes classification algorithm and a set of time-domain, user-dependent features. The system achieved an average specificity and sensitivity of 81.44 ± 8.35 and 82.30 ± 7.39%, respectively. The level of agreement between the intended and machine-predicted selections was moderate (κ = 0.60). The average information transfer rate was 0.87 bits/min with an average throughput of 0.31 ± 0.12 character/min. These findings suggest that an online TCD-BCI can achieve reasonable accuracies with an intuitive language task, but with modest throughput. Future interface and signal classification enhancements are required to improve communication rate.

## Introduction

Individuals who are cognitively aware but living with severe motor disabilities such as muscular dystrophy, multiple sclerosis, high-level spinal cord injuries or locked-in syndrome may not be able to use conventional means of expression such as speech and gestures for communication. Brain-computer interface (BCI) systems offer an alternative means of communication for these individuals (Tai et al., 2008). BCI systems enable users to generate a control command through mental activity alone (Tai et al., 2008). Many portable brain monitoring modalities have been explored for BCI development. The majority of systems have used electroencephalography (EEG) (Wolpaw et al., 2002), while hemodynamic-based monitoring modalities such as near-infrared spectroscopy (NIRS) (Sitaram et al., 2009; Falk et al., 2011), and transcranial Doppler (TCD) ultrasonography systems (Myrden et al., 2011) are emerging BCI alternatives. The cerebral hemodynamic response is inherently slower than the corresponding electrical response measured using EEG. In fact, there is a hemodynamic delay of 5–10 s between the onset of mental activation and the manifestation of blood flow velocity changes (Harders et al., 1989; Szirmai et al., 2005). However, hemodynamic monitoring systems are not prone to electro-genic artifacts due to muscle contractions or eye-movements. In particular, TCD-based systems have recently demonstrated high accuracies in offline studies (Myrden et al., 2011; Aleem and Chau, 2013).

TCD is a non-invasive ultrasound technology that detects the changes in cerebral blood flow velocity (CBFV). It was first introduced as a medical imaging device in 1982, and has been widely applied clinically (Aaslid et al., 1982) for the detection of increased intracranial pressure in neurocritical care, evaluation of subarachnoid haemorrhage, detection of microembolism, and monitoring of cerebral circulation during cardiopulmonary bypass (White and Venkatesh, 2006; Sarkar et al., 2007; Tsivgoulis et al., 2009; Reinsfelt et al., 2012).

TCD has recently been used as a functional brain imaging tool to examine the effects of mental tasks on the blood flow velocities. In particular, functional TCD studies have focused on the middle cerebral arteries (MCAs), which perfuse 80% of the brain, and thus measurements of velocities therein reflect cognitive effort levels (Vingerhoets and Stroobant, 1999; Stroobant and Vingerhoets, 2000). Blood flow lateralization elicited by mental tasks, such as verbal fluency and visuospatial tasks, has been detected using TCD in many studies (Aaslid, 1987; Vingerhoets and Stroobant, 1999; Stroobant and Vingerhoets, 2000; Haag et al., 2009; Whitehouse et al., 2009). Blood flow lateralization is due to the coupling between the cerebral blood flow and oxidative metabolism (Buxton and Lawrence, 1997). The left hemisphere of the brain exhibits augmented blood flow velocity during verbal fluency tasks while the right hemisphere demonstrates heightened activation during visuospatial tasks (Vingerhoets and Stroobant, 1999).

Recent functional TCD-BCI studies have reported promising rates of classifying different mental states (Myrden et al., 2011; Aleem and Chau, 2013; Faress and Chau, 2013). Myrden et al. (2011) first introduced TCD as a BCI measurement modality and discriminated between word generation and rest (average accuracy of 82.9 ± 10.5%) and between mental rotation and rest (85.7 ± 10%) in 9 able-bodied adults using 45 s task periods. The authors later followed up with a 3-class offline BCI, discerning among word generation, mental rotation and unconstrained rest with over 70% accuracy and reaching transmission rates of 1.2 bits per min (Myrden et al., 2012). Subsequently, in a study of 18 adults, Aleem and Chau (2013) reduced the task period to 18 s and classified successive left and right lateralizations offline in a user-independent framework with accuracies up to 74.6 ± 12.6%. Most recently, in an offline TCD-NIRS-BCI study, Faress and Chau (2013) achieved an average accuracy of 76.1 ± 9.9% in the automatic differentiation between pre- and post-verbal fluency hemodynamics (Faress and Chau, 2013). Collectively, these past offline TCD-BCI studies have shown that language (e.g., verbal fluency) and spatial tasks (e.g., mental rotation) elicit machine-discernible lateralizations in cerebral blood flow velocities in the MCAs, with time intervals as short as 18 s. The fundamental challenge of TCD-BCIs remains the relatively low throughput. Further, the viability of an online TCD-BCI has yet to be demonstrated.

In light of the above, the aim of the present study was to ascertain the achievable accuracy and throughput of communication with an online TCD-BCI. In particular, we implemented an online spelling system (i.e., scanning keyboard) controlled via two mental states, namely, rest and activation. The activation task was repetitive mental spelling and imagined writing of the intended word and the rest mental task was the visual tracking of a display of TCD signals. We hypothesized that previously reported offline accuracies in excess of 80% could be replicated in the online setting using an activation task that intuitively combined language processing and right-handed motor imagery.

## Methods

### Participants

Thirteen able-bodied participants were recruited for this study. Participants had normal or corrected to normal vision, and no reported history of neurological, metabolic, respiratory, cardiovascular, or drug/alcohol-related conditions. One participant was excluded after the first session due to the inability to accurately describe the study protocol. A second participant was excluded upon disclosing post-study, a medical history that violated inclusion criteria. A third participant was excluded due to inadequate transtemporal windows, which precluded the location of the MCAs. The ten remaining participants included for study (aged 18–40 years; all female), were all right-handed. All participants provided written informed consent. This study was approved by the research ethics boards of both Holland Bloorview Kids Rehabilitation Hospital and the University of Toronto.

### Instrumentation

**Figure 1:**
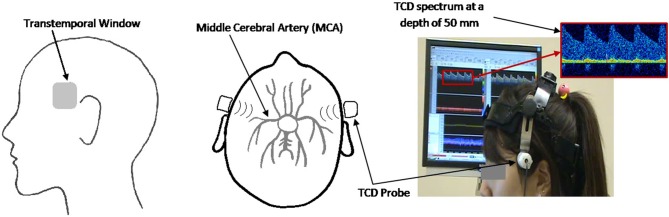
**Sagittal (left panel) and axial (middle panel) view of the ultrasound probe set at the transtemporal insonation window, directed toward the MCA**. Experimental setup (right panel) showing participant with TCD headgear and corresponding TCD spectrum.

The Doppler spectra of blood flow velocities through the left and right MCAs were monitored using the MultiDop X-4 TCD (Compumedics Germany) and the accompanying bilateral headgear with two fixed 2 MHz ultrasonic transducers. The data were recorded at a sampling frequency of 100 Hz. The probes were positioned over the transtemporal insonation window according to an established insonation procedure (Alexandrov et al., 2007) as seen in Figure 1.

Ultrasound gel was applied between the probe and the user's skin to ensure proper signal transduction. Once the probe was placed over the transtemporal window, the TCD was turned on with an initial depth setting of 50 mm. The insonation angle and depth were then adjusted to find the bifurcation of the internal carotid artery into the middle cerebral artery (blood flowing toward the probe) and the anterior cerebral artery (blood flowing away from the probe). The insonation depth was then decreased until the maximum unidirectional flow toward the probe was detected. All participants were given 5 min breaks per every 15 min of TCD usage to provide sufficient time for probe cooling. Throughout the recording process, the thermal cranial index (TIC) of the probes did not exceed 1.5, thus avoiding discomfort or thermal injury to the participants, which is in accordance with the British Medical Ultrasound Society safety guidelines (Group, 2010). The TCD device (MultiDop X-4) was approved by Health Canada's Medical Devices Directorate for investigational testing.

### Mental tasks

**Figure 2:**
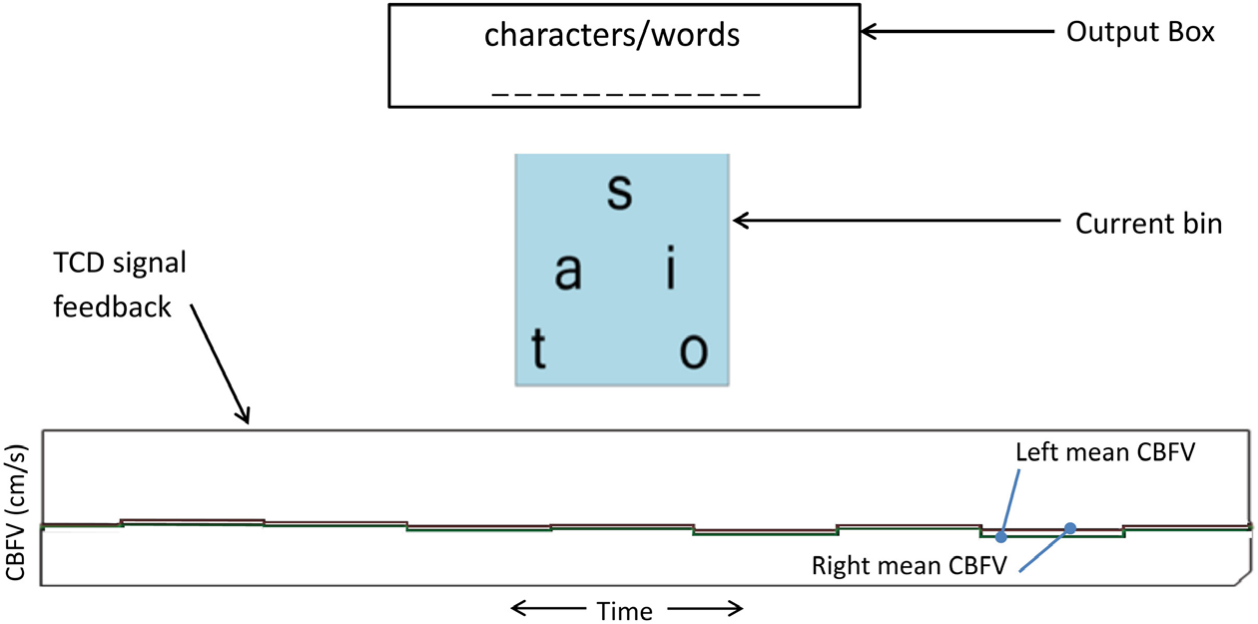
**TCD-BCI interface**. The bottom graph is the dynamic feedback signal showing a 10-s segment of left and right mean CBFV. Each step of the graph represents an average of 1.3 s of CBFV data (sampling rate of 100 Hz). This display facilitated the visual tracking task for inducing the rest state.

Participants performed two mental tasks (i.e., activation and rest) throughout the study. Mental spelling accompanied by imagined writing of each letter with the right hand was used as the activation task, with the intent of eliciting left-lateralized brain activity. To restore CBFV to non-lateralized basal levels, visual tracking of a time-evolving strip chart of left and right mean CBFV (Figure 2) was used as the rest task. The participant performed mental spelling throughout each 15 s activation period and the visual tracking task throughout each 15 s rest period.

During the training session, participants were presented with either a single letter or multiple letters forming part of a word. Upon seeing this cue, participants were instructed to repetitively rehearse the spelling of the desired word while simultaneously imagining the writing of the word with their right hand. Likewise, participants were instructed to shift their gaze to the TCD feedback signal whenever an hourglass appeared on the screen. Both tasks were completed without any vocalization to avoid an increase in blood flow due to speech.

In the testing sessions, the participants used the TCD BCI to spell target words online. During these sessions, the participants were asked to perform the activation mental task when the desired letter appeared among the currently available letter choices and to perform the rest mental task when the desired letter was not displayed.

### Dynamic keyboard

A custom on-screen keyboard was developed based on the concept of the dynamic keyboard developed by the University of Victoria. In our implementation, each level of the keyboard hierarchy contained multiple bins, although at any given time, only one bin was displayed to minimize mental workload and user confusion. Each bin contained multiple letters or words. Figure 2 depicts the user interface of the dynamic on-screen keyboard. The dynamic keyboard behaviors were governed by the following operating principles.

1. Letters are grouped into bins on the basis of their frequencies of use in the English language. For example, the initial letter bin contains the 5 most frequently occurring first letters (t, a, s, i, o) of English words. When one or more letters have been selected, subsequent letter bins contain the set of most probable next letters.
2. Whenever a letter bin is selected, a word bin containing the most frequent words starting with the sequence of letters selected thus far is presented.
3. Whenever a word bin is selected, each word within the bin is presented sequentially.
4. When none of the letters or words in a sequence is selected, the keyboard returns to the previous level of the hierarchy.
5. Whenever a selection is made, an “undo” option is immediately presented as a means of confirming the user's selection. The “undo” bin also provides an opportunity to delete the most recent letter or word, upon which the interface returns to the previous level of the hierarchy.

**Figure 3:**
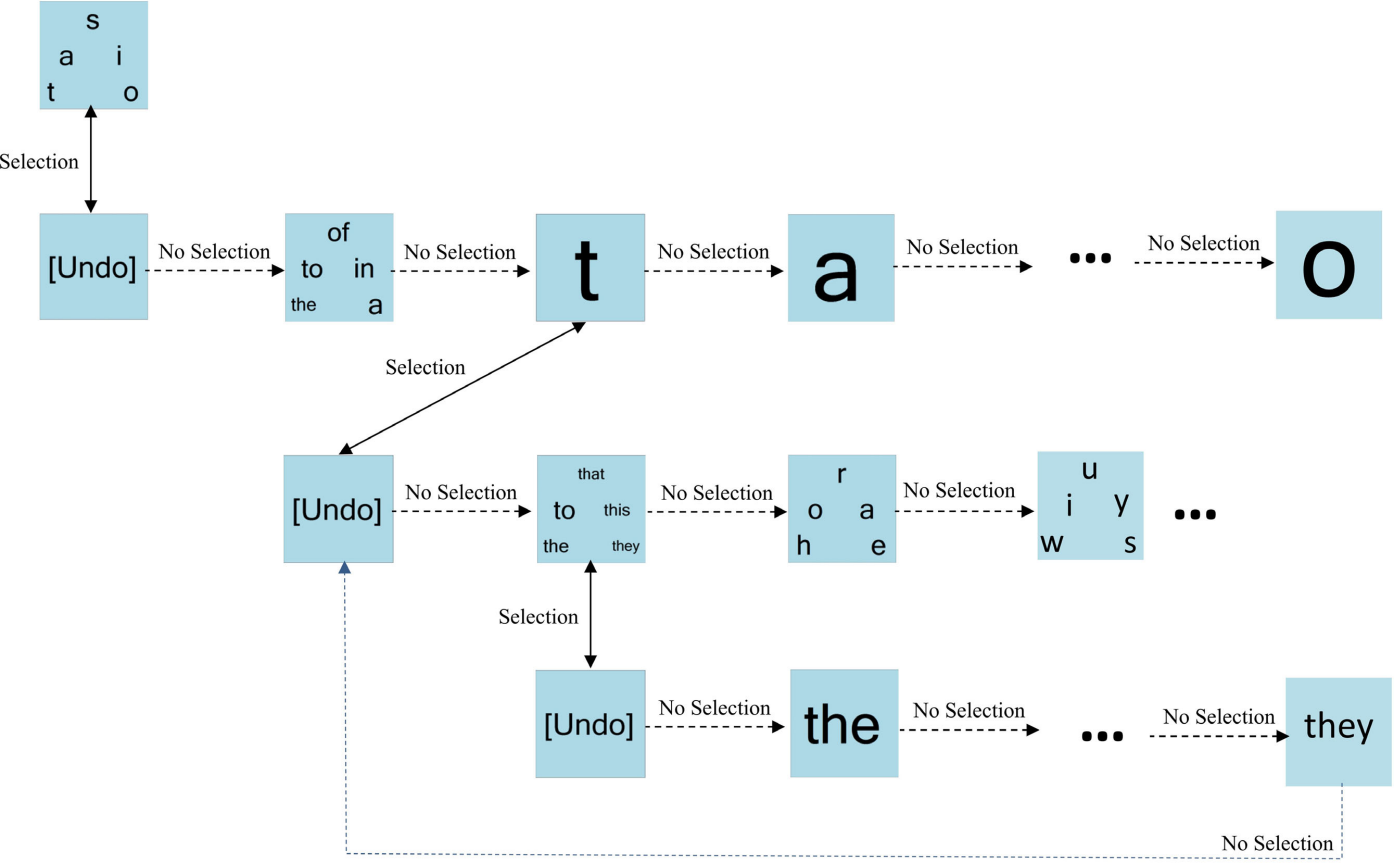
**An example of dynamic keyboard progression**. In this example, the user has selected the letter “t” and the bin containing high frequency words “the-to-that-this-they.”

Figure 3 portrays an example of dynamic keyboard progression. For simplicity, only a subset of paths is shown. Here, the bin “t, a, s, i, o” is selected. Bypassing the “undo” bin confirms the selection. The first bin on the next level of the hierarchy contains the highest frequency words starting with one of “t, a, s, i, o.” Here, this word bin is bypassed, triggering the presentation of individual letters from the previous level of the hierarchy. The letter “t” is chosen and confirmed (bypassing undo), prompting the presentation of high frequency words starting with “t.” In this example, this word bin is selected and confirmed, resulting in the presentation of the individual words from this bin.

### Experimental protocol

Each participant completed three sessions. At the beginning of the first session, each participant was given an information sheet, highlighting the nature of each task. In addition, prior to each session, participants also received verbal instruction about how to perform the activation and rest tasks. The first session involved two training blocks and one testing block while subsequent sessions contained one training block followed by two testing blocks. A one minute baseline recoding was obtained before each block for the purpose of normalizing data collected from the block. During baseline, participants performed the rest task. A five minute rest period was offered between blocks.

**Figure 4:**
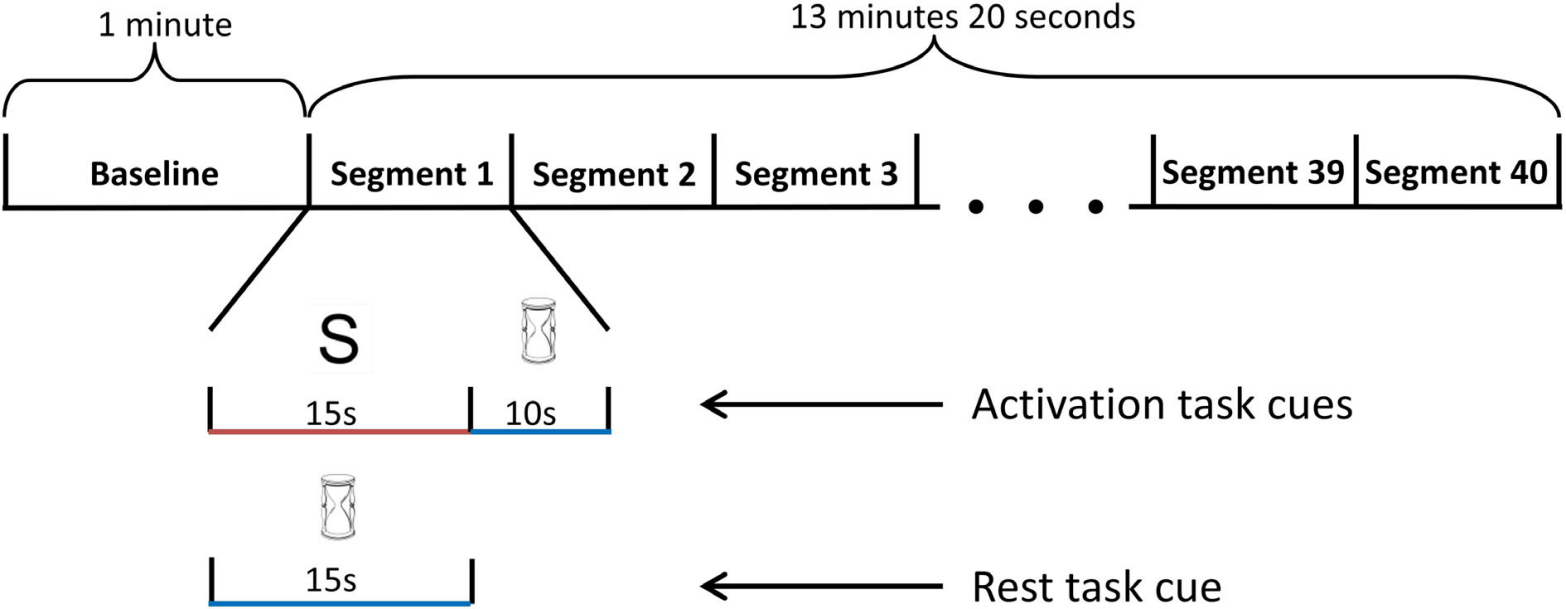
**Schematic diagram of the training block**. The training block began with a 1-minute baseline period, followed by 40 randomized task segments. During each task segment, the screen randomly displayed either an hourglass or a letter. If a letter was presented, the participant performed the activation mental task for 15 s, followed by the rest task (visual tracking of the TCD feedback signal) for 10 s. If an hourglass was displayed instead of a letter at the beginning of the segment, the participant continued to perform the rest task for an additional 15 s.

For each training block, the participants performed a total of forty task segments. Each segment was either an activation or rest task. The sequence of task presentation was randomized (Figure 4). A 10 s recovery period was included after each activation task to allow the participant's blood flow velocities to return to baseline levels. During the recovery period, the participants performed the rest task to restore basal blood flow velocities. In the first session, participants had a 10 min break while the two blocks of training data were used to train the appropriate classifier. For sessions two and three, the 10 min break occurred after the first training block. During this break, the classifier was trained with data from the current and initial sessions. After each session, the participants' level of fatigue was ascertained via a written survey.

For each testing block, the participants were asked to spell a given target phrase to the best of their abilities using the dynamic keyboard. The participants performed the activation task only when the bin containing the intended selection was presented. If a false positive occurred, the participants were instructed to select the undo button and correct the error before continuing the spelling process. If a false-negative occurred, the participants were instructed to simply wait until the keyboard looped back to the intended bin.

### Data processing and classification

**Figure 5:**
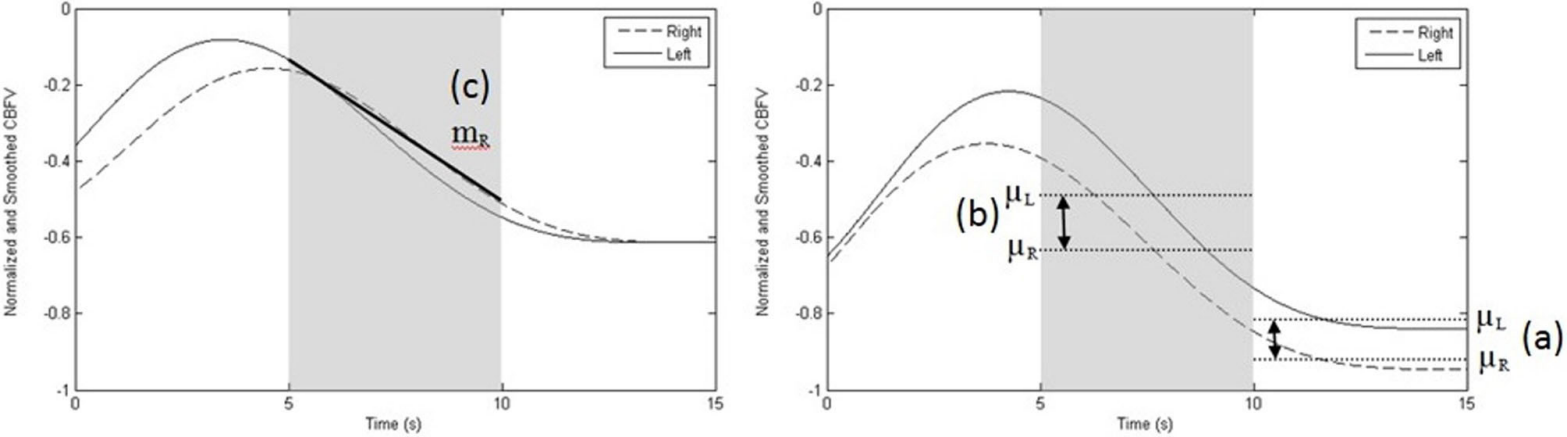
**Sample recording depicting the three most common features**. (a) difference between left and right mean velocities, μL–μR, at 10–15 s (right graph); (b) difference between left and right mean velocities, μL–μR, at 5–10 s (right graph), and, (c) slope of the right MCA CBFV (mR) at 5–10 s (left graph). Data shown are normalized and smoothed and represent one trial performed by participant 10. The left graph depicts a rest trial while the right graph portrays an activation trial, showing the difference between left and right mean CBFV at 5–10 s and at 10–15 s.

All data collected from the training blocks were used for classifier training. Therefore, for each participant, a total of forty activation data segments and forty rest data segments were used to train a user-specific classifier in session I. For each subsequent session, training data from session I (40 activation and 40 rest segments) and the session at hand (20 activation and 20 rest segments) were pooled for training (i.e., 60 activation and 60 rest data segments). Each segment was 15 s in duration. A total of forty-four features were extracted from each segment. Twenty four features were based on the left and right CBFV signal mean, slope, standard deviation, and entropy over the following intervals: 0–5, 5–10, and 10–15 s. Six features were extracted from the differences in mean and slope between the left and right signals over the same time intervals. Nine features were extracted based on the correlation, dot product, and mutual information between the left and right signals over the aforementioned time intervals. The last five features were extracted from left and right CBFV signal standard deviation and entropy from 0 to 15 s and the mutual information between the left and right CBFV signals over the 0 to 15 s interval. An example feature computation is presented in Figure 5. These features were chosen according to the findings of previous TCD brain lateralization studies (Myrden et al., 2011).

Weighted sequential feature selection (WSFS) was used to algorithmically select three to five features for each session, for each participant, to train a Naïve Bayes classifier. WSFS extended the sequential forward search (SFS) approach (Mamun et al., 2012) by explicitly considering feature contributions (i.e., the number of times a specific feature was chosen). For each fold of a 10-fold cross-validation for feature selection, all features were first ranked according to F-score (Duda et al., 2012) for interclass separability, using the training set. These ranked features were then organized into cumulative subsets such that the first subset contained the top ranked feature, the second subset contained the top two ranked features, and so on. The last subset contained all features. Within each fold, the subset with the highest validation accuracy was selected. Therefore, 10-fold cross-validation yielded 10 such subsets.

We enumerated the occurrence of each feature within these 10 subsets. Certain features appeared consistently across all subsets while others surfaced intermittently. The selected features were regrouped based on their frequency of occurrence, such that the *m*th group contained all the features that appeared at least *m* times, where *m* = {1, 2,…,10}. These new subsets were evaluated through a subsequent constrained 10-fold cross-validation (i.e., only the *m* pre-determined feature subsets were cross-validated) with newly randomized testing and training sets. The final set of features was then selected as that with the highest average validation accuracy. The chosen features were used to train a Gaussian Naïve Bayes classifier.

Figure 5 demonstrates a single trial of a rest task (left) and activation task (right). The three most common features selected across sessions and participants are highlighted. The least common of the three features (slope of the right MCA CBFV) was selected in six out of ten participants.

### Performance evaluation

To capture the different nuances of online classification performance, several metrics were invoked as suggested by Thomas et al. (2013) and Schlögl et al. (2007). To gauge the correctness of classification for a biased classifier (i.e., unequal performance for each class), sensitivity, and specificity were estimated from the confusion matrix Schlögl et al. (2007). Specificity is the number of true negatives divided by the actual number of negatives in the test set while sensitivity is the number of true positives divided by the actual number positives in the test set.

To measure the agreement between the predicted and desired selections (Cohen, 1960; Thomas et al., 2013) in the presence of unbalanced data (i.e., unequal number of samples per class due to the nature of the experiment), Cohen's kappa (κ) coefficient was estimated. Kappa ranges from 1 (perfect match) to 0 (chance level). If all values of κ within the 95% confidence interval around the mean are above 0 (κ ± 1.96 × φ(κ) > 0, where φ(k) is the standard error), then the average kappa value is significantly above chance (Friedrich et al., 2012). The classification accuracy ACC (overall agreement) was derived from the 2×2 confusion matrix *H*, as

(1) $$ACC=p_{0}=\frac{\sum_{i}H_{ii}}{N}$$

where *H*_*ii*_ are the main diagonal elements (i.e., number of correct classifications) of the confusion matrix *H* and *N* = ∑_*i*_∑_*j*_*H_ij_* is the total number of trials. The chance expected agreement *p_e_*, is the probability of observing the current confusion matrix and is given by,

(2) $$p_{e}=\frac{\sum_{i}n_{\text{+}i}n_{i\text{+}}}{N^{2}}$$

where *n*_+*i*_ and *n*_*i*+_ are the marginal column and row sums, respectively. The estimate of the kappa coefficient *κ* is thus,

(3) $$\kappa =\frac{p_{0}-p_{e}}{1-p_{e}}$$

while its standard error φ(κ) is given by,

(4) $$\varphi (\kappa )=\frac{\sqrt{p_{0}+p_{e}^{2}-\sum_{i}\left[n_{+i}n_{i+}\left(n_{+i}+n_{i+}\right)\right]/N^{3}}}{(1-p_{e})\sqrt{N}}$$

This method of evaluation is preferred for problems with unbalanced classes (Danker-Hopfe et al., 2004; Anderer et al., 2005), such as sleep classification.

To gauge performance of the system as a communication channel, we estimated the Nykopp information transfer rate (ITR), which is recommended for classification problems with unbalanced class sizes (Thomas et al., 2013). Letting *x*_*i*_ represent the actual input category (*x*_0_ = rest, *x*_1_ = activation) and *y*_*j*_ represent the predicted output (*y*_0_ = rest, *y*_1_ = activation), the ITR was given by

(5) $$ITR_{\text{Nykopp}}=\sum_{i\;=\;0}^{1}\sum_{j=0}^{1}p\left(x_{i}\right)p\left(y_{j}\;x_{i}\right)log_{2}\left[p\left(y_{j}\;x_{i}\right)\right]$$

where

(6) $$p\left(y_{j}\right)=\sum_{i=0}^{1}p\left(x_{i}\right)p\left(y_{j}\;x_{i}\right)$$

(7) $$p\left(y_{j}\;x_{i}\right)=\frac{H_{ij}}{n_{i\text{+}}}$$

while *p*(*x*_0_) = 0.7 and *p*(*x*_1_) = 0.3 are the prior probabilities of rest and activation tasks, respectively to be, estimated from the average frequency of occurrence of each task when spelling an intended message with no mistakes. To calculate the bit-rate, we multiplied the Nykopp ITR by the average number of trials per min (Thomas et al., 2013).

To assess system efficiency, the average throughput, defined as the number of characters output per min, was determined. Only correct characters were counted while the measured duration included the time required to make error corrections. Since participants were asked to correct mistakes during the spelling process, the estimated throughputs were generally conservative with the low char/min.

To measure the resemblance of the actual output to the intended output, the Levenshtein or edit distance was calculated. The edit distance compares the similarity between two strings of unequal length and is defined as the number of editorial operations required to convert the actual output into the intended output (Sankoff and Kruskal, 1993). Each deletion and insertion of a character was given a weight of 1 while a substitution was given a weight of 2, being equivalent to a deletion followed by an insertion (Sankoff and Kruskal, 1993). Since the intended outputs were of different lengths for the testing blocks of the three sessions, the edit distances were normalized based on the longest string length of the intended outputs (Equation 8). Other normalization methods more severely penalize a lack of input over an incorrect selection (Marzal and Vidal, 1993; Weigel and Fein, 1994; Li and Liu, 2007). However, due to the study design, an incorrect selection should have a higher edit distance than a lack of input since the effort required to correct an incorrect selection is far greater than that needed to produce an intended output with no corrections. The normalized edit distance, *D*_*EN*_, is given by,

(8) $$D_{EN}=\frac{D_{E}}{\left|X\right|}\times |X^{*}|$$

where |*X*| is the length of the intended output, *D*_*E*_ is the raw edit distance between intended and actual output, and |X^*^| is the length of the longest intended output from all sessions. Given the longest string length in our experiment was 18, i.e., |X^*^| = 18, a normalized edit distance of 18 indicated no output and 0 meant perfect match between the intended output and the actual output. Any score above 18 indicated that the actual output mismatched the intended output. The larger the normalized edit distance is, the further away the actual output was from the intended output.

## Results

### Feature selection

**Figure 6:**
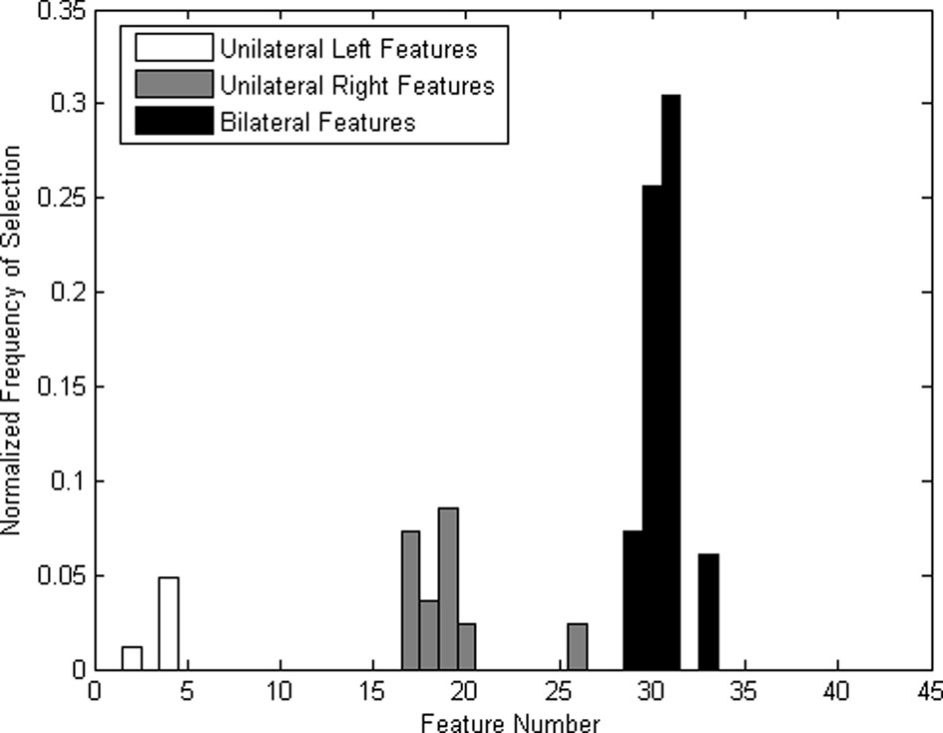
**Normalized frequency of features (the number of times a feature has been selected divided by the total number of times all feature have been selected) across all participants**.

Bilateral features were more frequently selected (Figure 6), which could be due to the left-lateralized nature of the language task. The higher selection frequency of bilateral features was consistent with that reported in a previous offline TCD-BCI study using verbal fluency (Myrden et al., 2011). Therefore, our modified verbal fluency task (i.e., rehearsing the spelling while imagining the writing of the target word) appeared to elicit machine-discernible left-hemispheric lateralization.

### Inter-participant analysis

The online performance on the testing blocks in sessions II and III is reported in Table 1. The system achieved an average specificity and sensitivity of 81.44 ± 8.35 and 82.30 ± 7.39% respectively, resulting in an average kappa coefficient of 0.60 ± 0.03. All participants exhibited a kappa coefficient that exceeded chance. Seven out of eight participants achieved a kappa coefficient over 0.4, which is equivalent to an accuracy >70% had the classes been balanced (Friedrich et al., 2012).

**Table 1:** **Classification performance within individual sessions**.

**Participant**	**Session**	**# Features selected**	**Specificity (%)**	**Sensitivity (%)**	**Kappa ***k*** ± φ**(*k*)****	**Information transfer rate *ITR*_Nykopp_ (bits/trial)**	**Bit-rate (bits/min)**
1	I	3	94.23	25.00	0.23 ± 0.19	0.05	0.16
	II	3	84.27	70.97	0.53 ± 0.14	0.21	0.65
	III	4	70.24	88.89	0.51 ± 0.13	0.23	0.73
2	I	1	78.26	71.43	0.43 ± 0.18	0.16	0.51
	II	3	75.00	86.11	0.54 ± 0.13	0.24	0.77
	III	2	82.93	94.74	0.72 ± 0.14	0.42	1.33
3	I	2	80.00	46.67	0.26 ± 0.17	0.05	0.16
	II	2	82.72	83.78	0.63 ± 0.14	0.30	0.93
	III	5	82.72	78.38	0.59 ± 0.14	0.25	0.78
4	I	2	32.65	72.73	0.03 ± 0.08	<0.01	0.01
	II	4	77.03	71.88	0.45 ± 0.14	0.15	0.49
	III	2	71.62	68.00	0.34 ± 0.13	0.10	0.31
5	I	4	36.84	100.00	0.27 ± 0.13	0.16	0.50
	II	1	83.13	91.89	0.69 ± 0.14	0.39	1.22
	III	1	71.80	90.91	0.56 ± 0.13	0.26	0.81
6	I	1	94.11	33.33	0.32 ± 0.20	0.09	0.27
	II	2	81.18	88.57	0.63 ± 0.14	0.33	1.03
	III	3	78.41	74.19	0.47 ± 0.13	0.18	0.57
7	I	4	88.89	71.43	0.59 ± 0.21	0.26	0.82
	II	3	90.91	71.88	0.63 ± 0.15	0.29	0.91
	III	3	93.26	80.65	0.74 ± 0.16	0.41	1.28
8	I	4	85.42	36.36	0.22 ± 0.17	0.04	0.13
	II	2	82.98	72.72	0.56 ± 0.16	0.21	0.66
	III	3	75.29	77.14	0.47 ± 0.13	0.18	0.56
9	I	4	82.00	40.00	0.20 ± 0.16	0.04	0.12
	II	3	88.64	93.75	0.76 ± 0.15	0.48	1.53
	III	3	86.05	81.82	0.64 ± 0.15	0.31	0.99
10	I	4	93.88	54.55	0.52 ± 0.22	0.20	0.64
	II	2	83.75	94.87	0.73 ± 0.15	0.43	1.37
	III	2	88.37	82.35	0.68 ± 0.15	0.35	1.10
Average online performance (sessions II and III)			81.44 ± 8.35	82.30 ± 7.39	0.60 ± 0.03	0.28	0.87

### Inter-session results

Classification performance across all sessions is summarized in Table 1. Only four out of ten participants were able to achieve above chance level kappa coefficient [κ ± 1.96 × φ(κ) > 0]. Of the four participants, three were able to achieve a moderate agreement within the first session (> 0.4). For sessions II and III, all participants achieved accuracies above chance. Moderate agreement between intended and predicted selections (> 0.4) was achieved in nine out of ten participants.

### Dynamic keyboard output and user feedback

**Figure 7:**
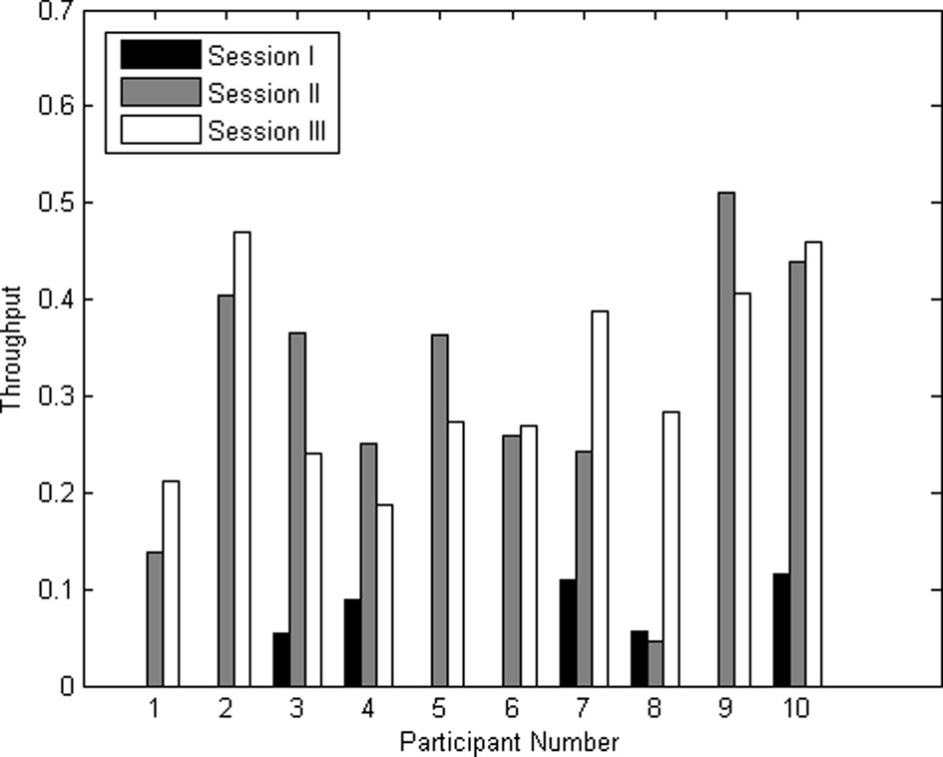
**Average throughput in characters/minute for sessions I, II, and III for all participants**.

The throughputs for all three sessions for all participants are shown in Figure 7. The average throughput for session I, II, and III across participants were 0.04 ± 0.05, 0.30 ± 0.14, and 0.32 ± 0.10 characters/minute, respectively.

**Figure 8:**
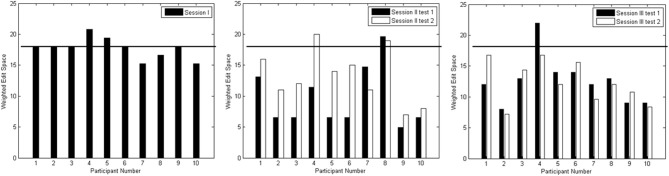
**Edit distances for test blocks from Sessions I (left plot), II (middle plot) and III (right plot)**. The horizontal line on each graph indicates an edit distance of 18 where no input was observed.

Figure 8 depicts the edit distances for each session. Using a paired *t*-test, we compared the edit distances for the 10 participants at a rigorous significance level of 0.01. In the session I test block, there was no significant difference between edit distances for the no output case (|X^*^| = 18) against distances when something was spelled (*p* = 0.619). In other words, the composed output was distant from the target output string. In session II, testing blocks 1 and 2 showed significant reduction in edit distances below that achieved in session I (*p* = 0.001; *p* = 0.005), though there was no significant difference between the edit distances of the two blocks (*p* = 0.019). In session III, testing blocks 1 and 2 again showed significant improvement over session I edit distances (*p* = 0.001; *p* < 0.001). In addition, there was no significant difference between edit distances of the two testing blocks in session III (*p* = 0.790). Finally, there was no significant difference between edit distances from the corresponding blocks of sessions II and III (*p* ≥ 0.114).

The correlation between tiredness levels and performance of all sessions were ascertained through Spearman's coefficient (*r*_*s*_) (Brown and Hollander, 1977). There were no significant correlations between the tiredness levels and edit space or throughput. In addition, there were no significant correlations between tiredness and specificity or sensitivity. However, there was a negative trend on the tiredness of the participant before the session and the specificity of the testing blocks (*r*_*s*_ = −0.314, *n* = 20, *p* = 0.177).

## Discussion

This study investigated the potential of controlling an onscreen keyboard via an integrated mental spelling-motor imagery activation task. Previous studies have demonstrated the potential of TCD as a BCI modality, but strictly in an offline setting (Myrden et al., 2011; Aleem and Chau, 2013; Faress and Chau, 2013). Using a mental spelling and motor imagery task for making selections we achieved online accuracies comparable to offline accuracies reported previously, but with modest throughputs.

### Throughput of the online TCD-based BCI communication system

The throughputs for sessions two and three improved beyond those of session one, approaching transmission rates of established BCI spelling devices (0.5 char/min) (Birbaumer et al., 1999). The observed combination of low throughput (Figure 7) and high kappa coefficient (Table 1) can be attributed to the cost (temporal penalty) of a false-negative. If a bin was unintentionally bypassed, the participant had to wait between 4 and 15 additional slides before the target bin would be presented again. This wait time can translate into a temporal penalty of several minutes for missing a selection, and is an inherent limitation of scanning keyboards. Additional practice may help to decrease response latency. Further, the Dynamic Keyboard interface could also be improved (e.g., context-specific word prediction) to enhance the speed and accuracy of letter/word selection.

### Feature selection

Some features were consistently selected across all participants. For most participants, the left lateralization of the mental task was pronounced. This finding confirms previous reports of left hemispheric lateralization accompanying verbal fluency tasks (Vingerhoets and Stroobant, 1999; Myrden et al., 2011). Due to the inherent lateralization, bilateral features were selected more frequently, as shown in Figure 6, particularly those corresponding to differences between the mean velocities of the left and right MCAs. Nine out of ten participants had the two most frequent features (i.e., difference of MCA means at 5–10 and 10–15 s) selected at least in one session. The difference in means between 10 and 15 s was the most frequently selected feature, followed by the difference in means between 5 and 10 s. The feature representing the difference in the means between 0 and 5 s was seldom selected. This is likely due to the inherent 5–10 s hemodynamic delay post-mental activation (Harders et al., 1989; Szirmai et al., 2005).

### Classification of mental spelling

All participants exhibited improved upon their session I performance in the latter 2 sessions. This improvement is attributable in part to the increase of training data available to the classifier. In addition, participants may have also become more familiar and comfortable with the study protocol and the user-interface. A longitudinal study of TCD-based BCI may help elucidate the effect of mental practice on functional performance.

Other factors (e.g., fatigue, extended trial duration or head motion) may have also impacted participant performance. For example, participant 4 reported a lack of concentration and physical fatigue, which may explain the lower accuracies for this individual.

### Communication rate

The TCD-BCI was able to achieve an average bit-rate of 0.87 bits/min and a maximum of 1.53 bits/min. If the post-activation task 10 s recovery time was removed, the average bit-rate would improve to 1.10 bits/min. In addition, if we are able to bring a three-task TCD into an online setting, similar to the offline study by Aleem and Chau (2013), assuming equal priors, we can further increase the bit-rate to 4.38 bits/min. Due to a lack of published online TCD-BCIs at present, we compare our results to those of other hemodynamic BCIs. Recent fMRI BCI studies using two-task algorithm attained an average of 2 bits/min (~80% accuracy). Other fMRI BCI studies with a four-task algorithm attained bit rates between 0.9 and 1.5 bits/min (~90% accuracies) (Yoo et al., 2004; LaConte et al., 2007; Minati et al., 2012). Thus, our system achieved a comparable bit-rate with a much simpler set-up. At present, EEG-BCIs still offer the most compelling bit-rates, typically in the order of 15–30 bits/min (Donchin and Arbel, 2009; Kansaku et al., 2010).

The average throughput for session I was not significantly different from 0 characters/min at a significance level of 0.01 (*p* = 0.022), which could have been due to the lowered specificity and sensitivity across participants in session I. Throughput for sessions II and III were significantly different from that of session I (*p* = 0.001; *p* < 0.001) and from 0 characters/minute (*p* < 0.001; *p* < 0.001). This improvement may be due, in part, to the user's increasing familiarity with the keyboard, facilitating more skilled navigation through the user interface. Given the temporal resolution of TCD and the sequential nature of the dynamic keyboard, the throughput may have approached its theoretical limit at 0.3 characters/min. Without modification of the user interface and the temporal window of data acquisition, further improvement of the throughput might not be possible. Incidentally, the change in throughput from session II to III was not significant (*p* = 0.653), but this does not preclude further improvements over extended periods of practice.

Similar to throughput, the edit distances for both session II and III improved significantly beyond session I values. Within sessions II and III, the edit distances for the actual outputs did not differ significantly. This suggested that the duration of TCD usage did not affect the quality of the output as testing block 2 typically occurred an hour after initial TCD set-up. Therefore, prolonged TCD usage may be possible provided that breaks are provided every 15~20 min.

### User feedback questionnaire

Feedback regarding the performance of the online TCD-BCI system was neutral to positive (except for the first sessions for participants 2 and 5). Participants 4 and 8 both indicated that they were “somewhat tired” prior to and “very tired” after every session. The lack of energy prior to the session may have impacted participant performance with the online TCD-BCI. The live feedback may have further frustrated the participants, exacerbating their fatigue and diminishing their concentration, thus forming a negative feedback loop that further impacted performance. However, the lack of significant overall correlation between tiredness levels and performance in terms of specificity, sensitivity, edit distance, and throughput suggest that user perceived fatigue did not directly impact overall user performance.

### Limitations

The inefficiency of the scanning keyboard undoubtedly constrained the observed BCI accuracies. Scanning keyboards are frequently used as an interface for assistive technology devices (Jans and Clark, 1994; Lesher et al., 1998). However, the existing keyboard interface was prone to long delays in the event of incorrect selections. For example, for individuals who achieved high accuracies (>85%), it was still difficult to spell the intended phrase within the allotted time. Further improvement of the Dynamic Keyboard is necessary to achieve more efficient communication in future studies. In addition, the required periodic cooling of the TCD probes introduces further delays in communication. Future improvements in TCD technology may minimize the required duration of probe cooling.

One of the major determinants for participant performance was their motivation and concentration. For participants who reported fatigue during specific sessions, (e.g., session 3, participant 4), the overall accuracy rates were lower compared to those of other participants. For participants who maintained concentration during the testing session, on the other hand, higher accuracies were observed (e.g., participant 7 and participant 9).

Despite the efforts to precisely locate the MCAs, unbalanced left and right CBFV magnitudes were occasionally observed. Probe placement errors may contribute to lower accuracies. Future TCD-BCI studies should endeavor to place the probes flush against the skin overlying the temporal bone and establish the same physiological insonation depth and sampling volume on either side of the head.

Another potential source of signal contamination for functional TCD studies is motion artifact. Conspicuous facial movements may shift the TCD probes, resulting in momentary or continuous deterioration of the recorded signals. Additionally, extensive body movements e.g., swinging of the arms, crossing and uncrossing the legs, and shifting body in the chair) may also introduce CBFV changes unrelated to the mental tasks at hand. Moderate movements (e.g., moving hands, and shifting feet) were observed in many participants during this experiment. However, the high classification accuracies achieved suggest a level of robustness to these motion artifacts.

### Future outlook

Compared to EEG, TCD is robust to electrical artifacts but, like near infrared spectroscopy, subject to long hemodynamic time constants which are several orders of magnitude greater than their corresponding electrical counterparts. However, unlike near-infrared spectroscopy BCIs, TCD is immune to ambient lighting. Based on these relative merits and the findings reported herein, TCD may fulfill a niche need where users possess sufficient literacy skills to do mental spelling, but may be unable to use electrical and optical alternatives, due for example to excessive myogenic noise or light absorption by dark hair. By addressing the aforementioned limitations, a TCD-BCI may eventually provide a means of bedside communication to non-verbal individuals who have severe motor impairments.

Future research will however need to go beyond the controlled, distraction-free laboratory conditions of the present study to gauge feasibility in realistic environments such as the home or inpatient unit. Further, future work must engage clients with physical disabilities to ascertain tolerance for the instrumentation and feasibility of the task paradigm.

## Conclusion

Using an online TCD-BCI system with an onscreen keyboard and combined mental spelling-motor imagery as the activation task, an average specificity, and sensitivity of 81.44 ± 8.35 and 82.30 ± 7.39%, respectively, were achieved with 10 able-bodied participants. The agreement between the intended and machine-predicted selections was moderate (κ = 0.60 ± 0.03), with an average information transfer rate of 0.87 bits/min. These results support further investigation of online bilateral TCD-BCI systems using intuitive language tasks.

### Conflict of interest statement

The authors declare that the research was conducted in the absence of any commercial or financial relationships that could be construed as a potential conflict of interest.
